# Effects of Heating Temperature on the Properties of Bio-Board Manufactured by Using Soybean Straw

**DOI:** 10.3390/ma13030662

**Published:** 2020-02-02

**Authors:** Xiaowen Song, Xiulun Wang, Koji Kito

**Affiliations:** Graduate School of Bioresources, Mie University, 1577 Kurimamachiya-cho, Tsu 5148507, Mie-Prefecture, Japan; 517D2S2@m.mie-u.ac.jp (X.S.); kitou@bio.mie-u.ac.jp (K.K.)

**Keywords:** soybean straw, bio-board, heating temperature, mechanical properties, dimensional stability performance

## Abstract

The objective of this paper is to effectively use soybean straw biomass resources and decrease the negative effects of using synthetic resin. Soybean straw was ground through a wet process then hot-pressed to make biodegradable fiberboard (bio-board) without any binder. The effect of heating temperature on mechanical properties and dimensional stability performance of produced bio-board was investigated. Bonding quality and chemical changes of the bio-board were also evaluated using scanning electron microscopy (SEM) and Fourier transform infrared (FTIR) spectroscopy. The moisture content decreased from 12.5% to 3.4% with the increase of heating temperature. Meanwhile, most mechanical properties of bio-board improved. However, an excessive heating temperature, especially at 230 °C, did not significantly promote the improvement of most mechanical properties. However, the dimensional stability performance of the bio-board was greatly improved from 140 °C to 230 °C. Overall, the results showed that bio-board could be made by using soybean straw without any synthetic resin. Heating temperature plays a significant role in affecting the properties of bio-board. The refined bio-board is expected to be used as a packaging material, heat insulation in architecture, and mulch film for agricultural purposes.

## 1. Introduction

Conventional fiberboard is mostly composed of wood cellulose combined with petroleum-based resins. As the board usually has a flat appearance, even texture, and excellent machining performance, it is widely used in furniture, packaging, and construction industries [1]. Therefore, the use of fiberboard has greatly increased because of infrastructure needs in developing countries in recent years. Consequently, the vast consumption of wood causes tremendous pressure on the supply of timber, and influences global forest resources. The other critical issue is toxic substances released in the use of fiberboard. Toxic substances, such as formaldehyde gases, are harmful to the health of the human body and unfriendly to the environment, which motivates human beings to realize the importance of the sustainable development of resources and the environment. Given this, it is worthwhile to use other annually grown plant biomass to produce non-toxic, biodegradable fiberboard [2].

As an important source of staple food and oil-bearing crops, approximately 352.6 million tons of soybeans and the equivalent amount of its straw were produced globally in 2017. The straw of soy is widely available, inexpensive, and annually renewable [3]. However, because of the lack of a comprehensive regulatory policy to manage straw resources, most of the straw is discarded or burned. This not only causes severe air pollution but also wastes biomass resources. The regular composition of soybean straw includes 35% cellulose, 21% insoluble lignin, 17% hemicelluloses, 11% ash, 1% acid-soluble lignin, and 5.8% extractives [4]. Reddy et al. [5] indicated that technical fibers extracted from soybean straw had good qualities, which were suitable to be used in composite materials and other industrial applications. Nevertheless, few reports are available regarding the field of studying board manufacturing using soybean straw.

The other urgent issue regards the release of toxic formaldehyde gas during the application of fiberboard in daily life. Binderless board is beneficial, being manufactured by relying on the self-bonding of the internal composition of raw materials instead of using synthetic binders to solve this problem. It was reported by Hubbe that the inter-fiber bonds such as hydrogen bonding between fibers and dispersion force were the main factors influencing the properties of panels rather than individual fiber strength [6]. Chemical pretreatment has been applied for facilitating the adhesive ability among fibers. Alkaline [7], acid [8], and oxidation agents [9] are commonly used as chemical agent. Halvarsson et al. [10] made fiberboard by using wheat straw fiber that was treated through Fenton’s reagent. The result showed that the mechanical properties were enhanced with the increase of hydrogen peroxide. Li et al. [11] evaluated some mechanical and physical properties of MDF (Medium Density Fibreboard) whose raw materials were pretreated with oxalic acid. While this treatment increased the water resistance to a certain extent and was beneficial for the lightness of board, there was no promotion for properties of internal bond strength of the panel. Enzymatic pretreatments were also used for promoting fiber bonding [12]. Laccase is a kind of polyphenol oxidase containing copper, which is found in fungi, higher plants, bacteria and some insects [13]. It was reported by Nasir et al. [14], who used SEM to observe a fiber surface, which was pretreated by laccase. The morphology manifested a smooth fiber surface covered with a uniform layer of softened lignin, which enhanced the fiber bonding ability. Whether it was chemical pretreatment or enzymatic pretreatment, reaction time, and reagent concentration shall be controlled to avoid excessive removal of lignin and decomposition of cellulose. At the same time, these two methods have a limited effect on improving dimensional stability.

Lignin holds a large proportion of vascular plants and some algae. It acts as a binder and reinforcement in the cell wall formation [15]. Therefore, the addition of lignin and full use of lignin as an intermediary for fiber bonding is expected to enhance the properties of non-resin board. Anglès et al. [16] evaluated the effect of different components of lignin on the properties of the board, which proved that water stability and mechanical properties were noticeably increased. Domínguez-Robles [17] also explored the different percentage of lignin content on mechanical properties of wheat straw board, and results showed that the mechanical properties were proportional to the lignin content (from 0% to 15%).

Furthermore, Zhou et al. [18] manufactured binderless fiberboard by using cotton stalk with enzymatic hydrolysis lignin, and physico-mechanical property tests were carried out. It turned out that the self-bond ability among fibers increased with the softening of lignin, which was closely related to fiber moisture content and pressing temperature. Okuda et al. [19] produced binderless board using kenaf core, and explored the physical properties and chemical changes during the hot-press stage. The results showed both the softening of lignin and condensation reactions of lignin took place during this period. These changes were in favor of enhancing the fiber self-bonding ability. Heating temperature seems to be the most influential parameter of lignin changes, subsequently affecting the properties of binderless board. A similar conclusion has been reported by Hashim et al. [20], Salvadó et al. [21], and Wang et al. [22].

In this paper, as an annual planting source, soybean straw was ground through a wet process and molded by a hot-press device to make biodegradable fiberboard (bio-board). To increase the self-bonding ability among fibers, and improve the properties of bio-board, it is necessary to evaluate the properties of bio-board made from soybean straw under different heating temperature. The mechanical properties of the bio-board, including bending strength, tensile strength, screw-holding force, and internal bond strength, were investigated. Additionally, the dimensional stability performance, measured by thickness swelling (TS) and water absorption (WA), was evaluated. Furthermore, thermal gravimetric analysis, Fourier transform infrared (FTIR) spectroscopy, and scanning electron microscopy (SEM) were used to investigate the thermal stability of raw materials, chemical changes, and microstructural changes corresponding to different heating temperatures.

## 2. Materials and Methods 

### 2.1. The Process of Manufacturing Board

**Figure 1:**
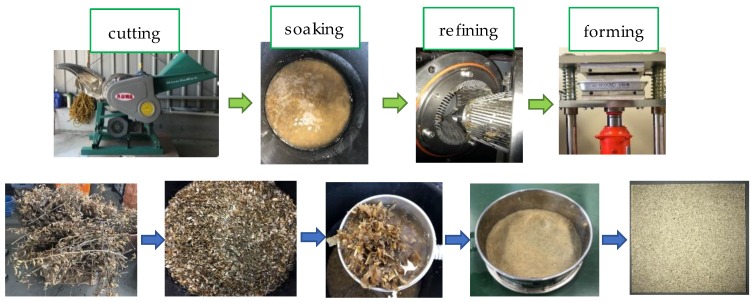
The making process of bio-board.

The procedure of manufacturing bio-board, including cutting, soaking, refining, and forming, is shown in Figure 1. First, the soybean straw was cut by an electric disintegrator (SU16, CO WA cutter Corp., Mishima, Shizuoka, Japan) into chips shorter than 10 mm. After that, the tiny chips were immersed in water at 20 °C for 96 h. In the process of soaking, the fibers were softened and swelled gradually. This process was beneficial for refining treatment, which converted soybean straw into pulp. In the refining process, the soaked soybean straw was fibrillated by a beat refiner (Model A Beat finer, Satomi Corp., Shizuoka, Japan). The fiberization of soybean straw was conducted by using a waterway circulation system of the refiner, in which the straw, along with running water, passed through rotating blades repeatedly. In the end, the soybean straw became tiny fibers, and the generated fiber pulp could pass through the sieve of 2 mm.

**Figure 2:**
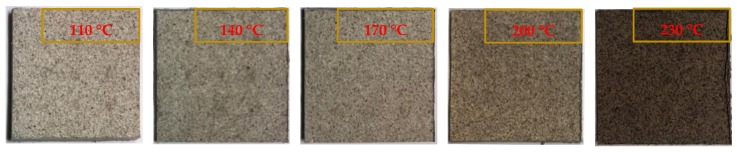
Bio-board produced at different heating temperature.

The bio-board was manufactured using a hot press (IMC-180C Imoto Corp., Kyoto, Japan) with a manually controlled hydraulic press system. The heating temperature of hot-press was controlled by the PID method, and the maximum heating temperature was up to 340 °C. The applied pressure adjusted from 0 MPa to 12.4 MPa. In the process of forming, 15 pieces of bio-boards in five categories were made in several conditions. There was increase of temperature from 110 °C to 230 °C at intervals of 30 °C. The condition of pressure was 5 MPa, which was accepted to be the optimum in some preliminary tests. The time of pressing and heating was 30 min in the process of forming. The pre-pressing was carried out first. The evenly distributed 500 mL fiber pulps were slowly poured into a mold with dimensions of 100 mm × 100 mm × 40 mm and pre-pressed at 5 MPa in room temperature. During this stage, excess water squeezed out, and the pre-pressed mat was shaped. Then, the pressure and temperature applied on the metal mold was controlled by the hot-press machine. The produced bio-boards are shown in Figure 2. They exhibited smooth surfaces and colors ranging from light brown to dark brown. Furthermore, they were conditioned at a constant humidity and room temperature more than three days before the measurements and tests were conducted.

### 2.2. Thermogravimetric Analysis

The thermogravimetric analyses (TGA) of the raw materials were conducted using TA Q500 (TA Instruments, New Castle, DE, USA) thermogravimetric analyzer. Twenty milligrams of dried soybean straw fiber were placed into the alumina crucible of the TA Q500 and heated from 25 °C to 700 °C at a heating rate of 20 °C min^−1^ under a nitrogen atmosphere.

### 2.3. Spectroscopic Analysis

FTIR spectroscopy was used to characterize the type of functional groups existing in samples from produced bio-boards. Sample pellets were mixed containing 5 mg of bio-board powder and 95 mg of finely ground potassium bromide (KBR), which was then pressed into pellets lower than 1 mm in thickness. After that, the prepared pellets were investigated with a Nicolet 380 FTIR spectrometer (Thermo Fisher Scientific Corp., Waltham, MA, USA) between 4000 cm^−1^ and 400 cm^−1^ with a resolution of 2 cm^−1^.

### 2.4. Scanning Electron Microscopy Analysis

SEM analysis was used to conduct the cross-section observation related to the fiber bonding quality inside bio-board. The SEM samples with a dimension of 0.5 cm × 0.5 cm were cut from bio-boards using an ultrasonic cutter (ZO-40B Honda Plus+ Corp., Shinshiro, Aichi, Japan), in which a smooth cross-section surface could be generated. Then, the samples were coated with gold using the field emission Inspect SEM (Inspect F50, FEI Corp., Hillsboro, OR, USA). Furthermore, the microstructure of bio-board was observed with FEI Inspect at 10.00 kV in Secondary Electron Imaging (SEI) mode.

### 2.5. The Test for Mechanical Properties

**Figure 3:**
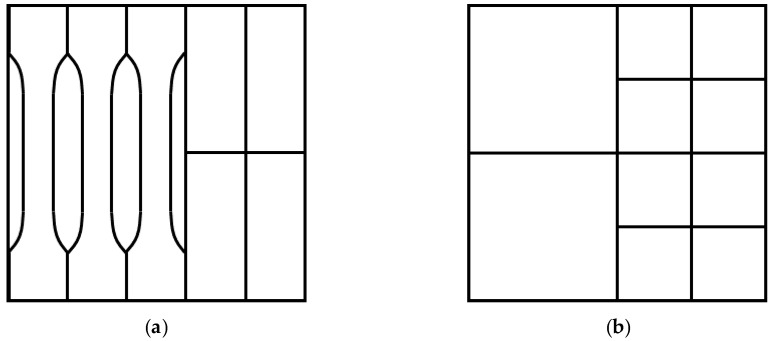
The specimens from bio-board: (**a**) specimens for bending test (50 mm × 20 mm) and tensile test (100 mm); (**b**) specimens for screw-holding force test (25 mm × 25 mm), internal bond strength test (25 mm × 25 mm), and dimensional stability test (50 mm × 50 mm).

The densities were measured for each bio-board by means of dividing the bio-board’s mass to its volume. After that, the bio-boards were divided into four specimens for bending strength test and three specimens for tensile strength test, as illustrated in Figure 3a. Other bio-boards were divided into specimens for screw-holding force test, internal bond strength test, and dimensional stability test as described in the next section and Figure 3b. The above-mentioned mechanical properties and dimensional stability performance of bio-board were evaluated according to Japanese Industrial Standard JIS-A5908 [23].

The mechanical property tests were conducted with a universal material testing machine (SVZ-200NB-200R2, IMADA Corp., Toyohashi, Aichi, Japan). The instrument has automatic compensation function. The measurable load was in the range of 20 N to 2000 N. The resolution of load measurement was 0.1 N, and displacement measurement resolution was 0.01 mm. The dumbbell-shaped specimens were used for tensile tests at a test speed of 10 mm/min. Tensile rupture stress was calculated by using Equation (1). Bending rupture stress was obtained from specimens which had dimensions of 50 mm × 20 mm. The specimens were loaded with a central loading nose and two lower supports, each with a radius of 5 mm, and the distance between the two lower supports were adjusted and set to 40 mm. During the test, the load was applied with test speed of 10 mm/min. Bending rupture stress was calculated using Equations (1) and (2).

(1) $$\sigma_{t}=\frac{F}{\mathrm{bh}},$$

(2) $$\sigma_{b}=\frac{3\mathrm{Fl}}{2{\mathrm{bh}}^{2}},$$

where σ_t_ is tensile stress, σ_b_ is bending stress, F is applied load, l is supported span, b is width of specimen, and h is thickness of specimen.

After the bending test and the tensile test, the specimens of two tests were used to calculate the moisture content. Samples of different conditions were placed into aluminum boxes, separately. Then the moisture of specimens was evaporated in a dry oven (LDO-450S, IUCHI Corp., Tokushima, Japan) with a temperature of 110 °C for 24 h. Moisture content was calculated by using Equation (3).

(3) $$\mathrm{MC}=\frac{m_{a}-m_{b}}{m_{a}-m_{c}},$$

where MC is moisture content, m_a_ is mass of samples before evaporation, m_b_ is mass of samples after evaporation, and m_c_ is mass of empty aluminum box.

Internal bond strength (IB) was measured by pulling the specimens apart perpendicular to the side surface. First, the twin side surface of specimen (25 mm × 25 mm) was bonded to the aluminum blocks with strong double-sided tape (SPS-25, 3M Scotch Corp., St. Paul, MN, USA). Then the load was applied to the aluminum block with a speed of 2.4 mm/min. The maximum load was obtained before the test piece was torn off, which was used to calculate the IB strength by using Equation (4).

According to the provisions of JIS-A5908, the screw-holding force test was carried out by using the screw with 2.7 mm in diameter, and the screwed depth was 11 mm. Owing to the limitation of dimensions of mold, it was hard to make a bio-board with a thickness greater than 11 mm in this experiment. Based on the JIS-A5908 standard, after trials and tries, when the thickness was less than 11 mm, the calculation formula of the screw-holding force was summarized as Equations (4) and (5).

(4) $$\mathrm{IB}=\frac{F_{\max}}{a^{2}},$$

(5) $$\mathrm{SHF}=\frac{{\mathrm{CF}}_{\max}}{\mathrm{hd}},$$

where F_max_ is the maximum load, a is side length of specimen, h is thickness of specimen, d is the nominal diameter of wood screw, and C is correction factor compared to the standard experiment, which is 25.155.

### 2.6. The Test for Dimensional Stability 

WA and TS were measured to evaluate the dimensional stability of bio-board. The 50 mm × 50 mm specimens were soaked in water at the depth of about 2 cm below the water surface, then placed in an incubator with the temperature of 20 ± 1 °C for 24 h. Mass and thickness of bio-board samples measured before and after soaking were used to calculate WA and TS as described in Equations (6) and (7).

(6) $$\mathrm{WA}=\frac{m_{2}-m_{1}}{m_{1}},$$

(7) $$\mathrm{TS}=\frac{t_{2}-t_{1}}{t_{1}},$$

where m_1_ is mass before immersion, m_2_ is mass after immersion, t_1_ is thickness before immersion, and t_2_ is thickness after immersion.

### 2.7. Statistical Analysis

All data reported were the average values of duplicates. Experimental data were evaluated by one-way analysis of variance (ANOVA), and *p* < 0.05 was considered statistically significant.

## 3. Results and Discussion

### 3.1. Thermal Properties

**Figure 4:**
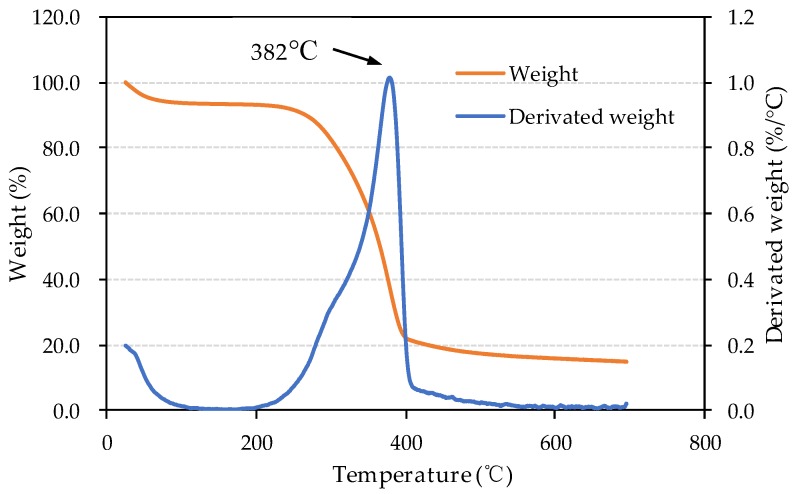
TG and DTG curves of the samples.

TGA results of bio-board are shown in Figure 4, which represents weight loss curves (TG) and derivative thermogravimetric (DTG) curves. The TG curves show an initial decrease in weight while the temperature is lower than 110 °C. This weight loss could be caused by moisture in the soybean straw powder diffused in the air by heat. After that, the weight does not change substantially between 110 °C and 200 °C. From 200 °C to 400 °C, the weight decreases from 93.1% to 22.2%, and derivate weight loss rate achieved the maximum value at a temperature of 382 °C. Macedo et al. [24] reported the sequence of thermal degradation of the plant biomacromolecules was hemicellulose (200–260 °C), cellulose (240–350 °C), and lignin (280–500 °C). The thermal decomposition of hemicellulose was expected to occur in the process of production of bio-board at 200 °C and 230 °C. When the temperature is above 400 °C, the rest of the lignin tends to gradually degrade until about 14% of the ash was left.

### 3.2. Functional Group Analysis

**Figure 5:**
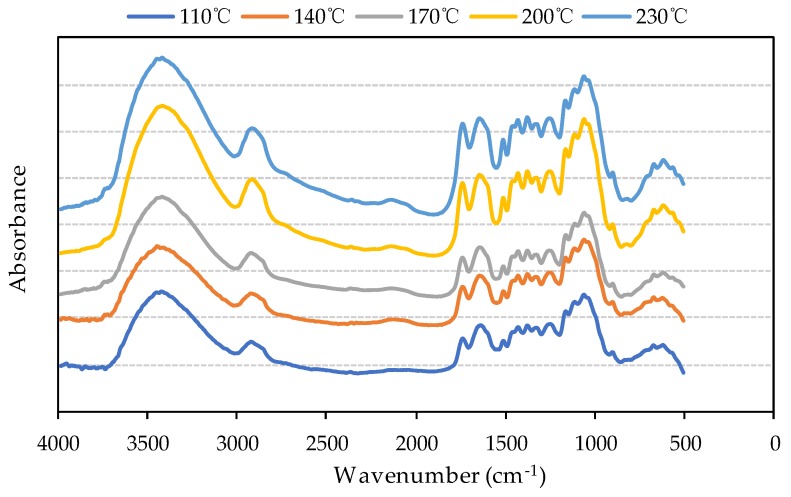
FTIR spectra of the samples at different heating temperature.

The FTIR spectra of the produced bio-board at different heating temperatures are shown in Figure 5. The samples of bio-board do not have significant difference in accordance with the peak position of the spectrum. The absorption band around the 3400 cm^−1^ indicates –OH stretching. It indicates the existence of hydrogen bonds or –OH in aliphatic aromatic compounds of the samples [25]. The peak around the 2900 cm^−1^ is related to the C–H stretching vibration. Two peaks are generated between 1680 cm^−1^ and 1750 cm^−1^ which might be caused by the conjugating of the benzene ring with the hydroxyl (–OH) or amino group (–NH_2_), resulting in a ring-absorbing peak [26].

A series of peaks are generated between 1000 cm^−1^ and 1500 cm^−1^ which is associated with the stretching vibration of C–O, C–C, and the bending vibration of C–OH. It is worth noting that the absorbance at 3400 cm^−1^ at the temperature of 200 °C and 230 °C is higher than that at other temperatures. The results could be attributed to the degradation of hemicellulose as reported by Bledzki et al. [27,28]. Because hemicellulose was a hydrophilic substance, the reduction of it could be potential evidence to the highly waterproof performance of the bio-board made at high temperature.

### 3.3. SEM Analysis

**Figure 6:**
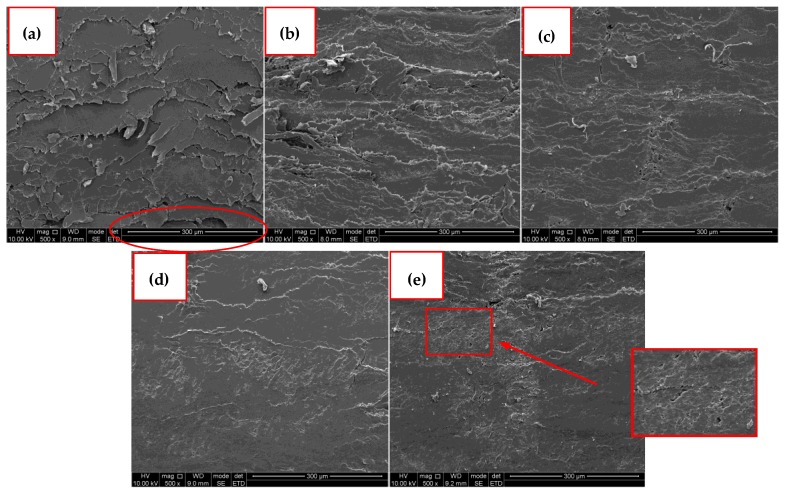
Section morphology of bio-board at different heating temperature (×500). (**a**) 110 °C; (**b**) 140 °C; (**c**) 170 °C; (**d**) 200 °C; (**e**) 230 °C.

The section morphology of the bio-board manufactured from 110 °C to 230 °C is shown in Figure 6. In Figure 6a, the section of the bio-board made at 110 °C showed continuous narrow irregular fissure existing among the observed zone clearly, and some scaly shape covers the surface. Moreover, uneven delamination means a heterogeneous fiber distribution inside the bio-board, and this might bring about poor mechanical performance externally. With the increase of heating temperature, the cross-section texture becomes dense and uniform. When the temperature reaches 200 °C, it exhibits a smoother cross-section texture in Figure 6d, which implies a superior mechanical property. However, at 230 °C in Figure 6e, there exist holes and signs of thermal degradation in the cross-section, indicating that the excessively high temperature is less promoted for the improvement of the mechanical properties.

### 3.4. The Physical Properties of Bio-Board

**Figure 7:**
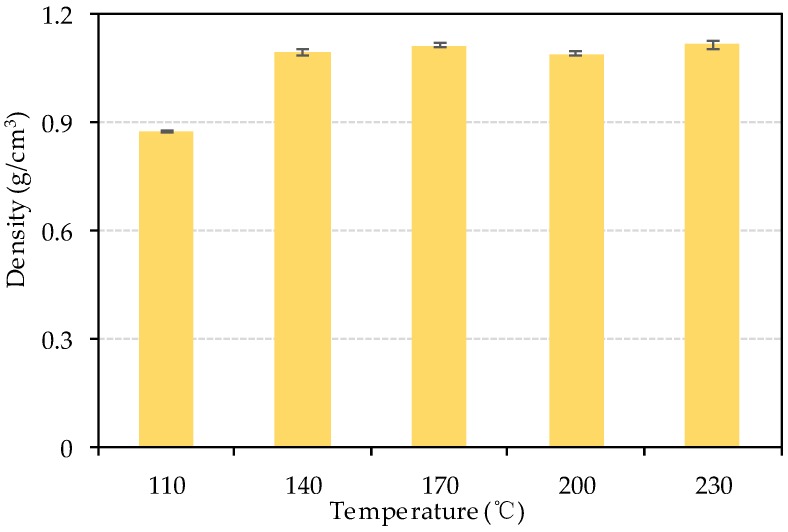
Density of bio-board at different temperature.

Figure 7 shows the density of the bio-board produced in this study. The density at different heating temperature is almost the same as 1.1 g/cm^3^, except for the density 0.88 g/cm^3^ at 110 °C. These bio-boards are classified as hard board according to JIS A5908 because the density exceeds 0.8 g/cm^3^.

**Figure 8:**
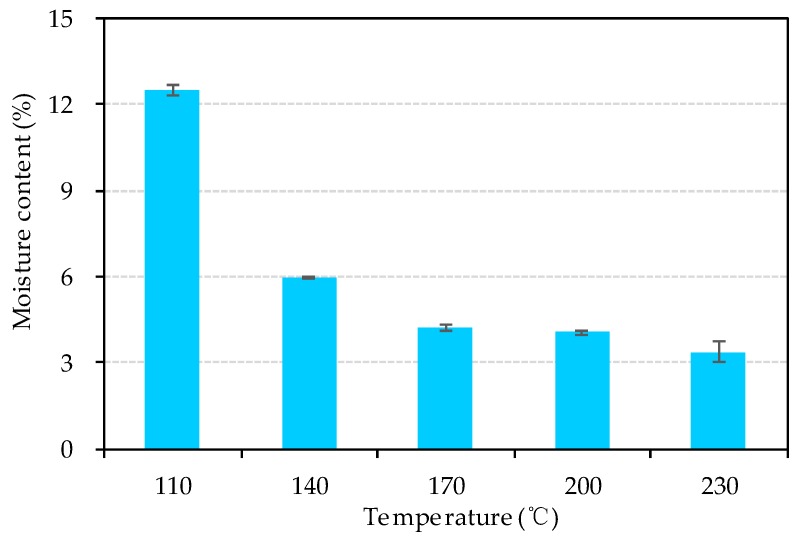
Moisture content of bio-board at different temperature.

Figure 8 exhibits the moisture content of bio-board at each heating temperature. It is obvious that the moisture content decreases with the rise of temperature. When the temperature is 110 °C, the moisture content is 12.5% larger than that of others. It could be inferred that there are many tiny voids filled with air and free water molecules inside the bio-board made at 110 °C. The SEM results also show that there exist some fissure in the samples. Therefore, the density of bio-board made at 110 °C is lower than the others. Similar results were also reported by Ramos et al. [29].

### 3.5. The Mechanical Properties of Bio-Board

**Figure 9:**
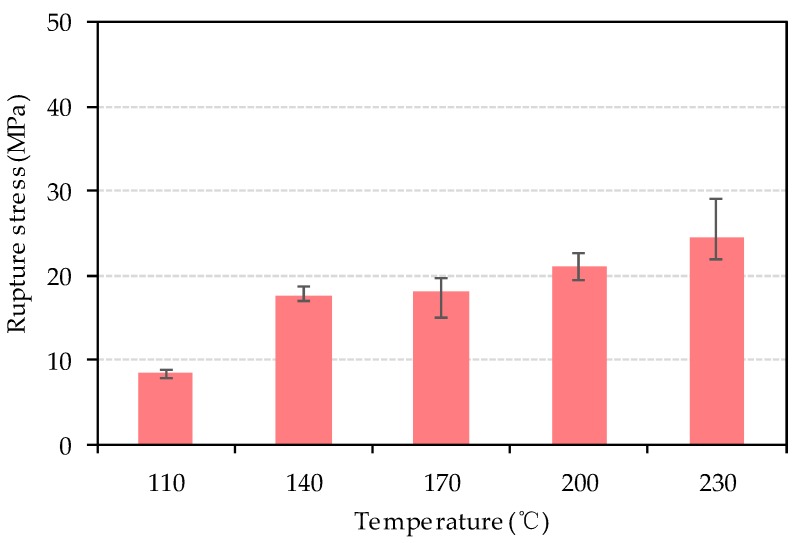
Tensile rupture stress of bio-board at different temperature (*p* < 0.05).

**Figure 10:**
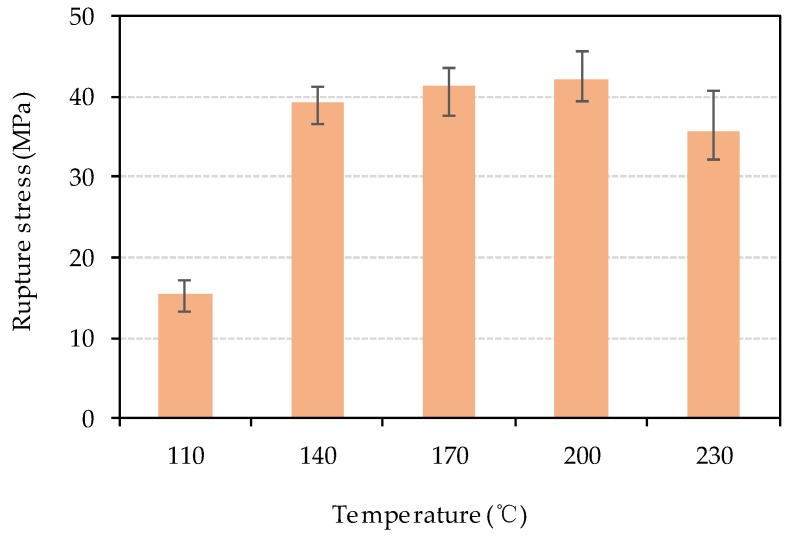
Bending rupture stress of bio-board at different temperature (*p* < 0.05).

To investigate the strength of bio-board produced in this study, a bending test and tensile test were carried out. Figure 9 and Figure 10 represent the tensile rupture stress and the bending rupture stress of bio-board, respectively. As shown in Figure 9, the tensile rupture stress is between 8.4 MPa and 24.4 MPa, increasing with the rise of heating temperature. As shown in Figure 10, the bending rupture stress of bio-board produced at 110 °C is 15.5 MPa which was significantly lower than the others. From 140 °C to 230 °C, the bending rupture stress first experienced a slow increase from 39.3 MPa to 42.1 MPa and dropped to 35.8 MPa at 230 °C. The JIS A5908 hardboard S35 type required minimum bending rupture stress of 35 MPa. Nearly all the bio-board met the requirement for bending rupture stress, except the bio-board made at 110 °C.

According to the moisture content discussed above, it is clear that the higher water content was the reason that both bending and tensile strength of the bio-board pressed at 110 °C had lower rupture stress. From 140 °C to 200 °C, the moisture content of bio-board decreased as shown in Figure 8, and the reduction of free water molecules represented the direct connection among fiber macromolecules through hydrogen bonding, or van der Waals forces were enhanced. Moreover, a softening of lignin during the forming process also occurred, and it was beneficial for bonding the fiber together more tightly. The lignin glass transition temperature was directly related to the moisture content according to the previous literature [30,31]. The wet-forming method was used in this study. In the process of forming, the high moisture content of the bio-board mat would significantly reduce the glass transition temperature of lignin, and the softened lignin acted as a binder. Therefore, the bending strength and tensile strength slightly increased. Bio-board produced at 230 °C had a minimum moisture content of 3.4%, as shown in Figure 8. However, it is worth noting that the average tensile rupture stress increased by 16.5% compared with that of 200 °C, but the average bending rupture stress decreased by 15.1%. The results might be related to the chemical changes of material. According to Macedo et al. [24], the hemicellulose would be pyrolyzed above 200 °C, and the pyrolysis products underwent condensation or polymerization with the temperature rise. At 230 °C, the condensation reaction and plasticization brought about an embrittling and hardening of the bio-board, which was not favorable to the bending strength of the bio-board.

**Figure 11:**
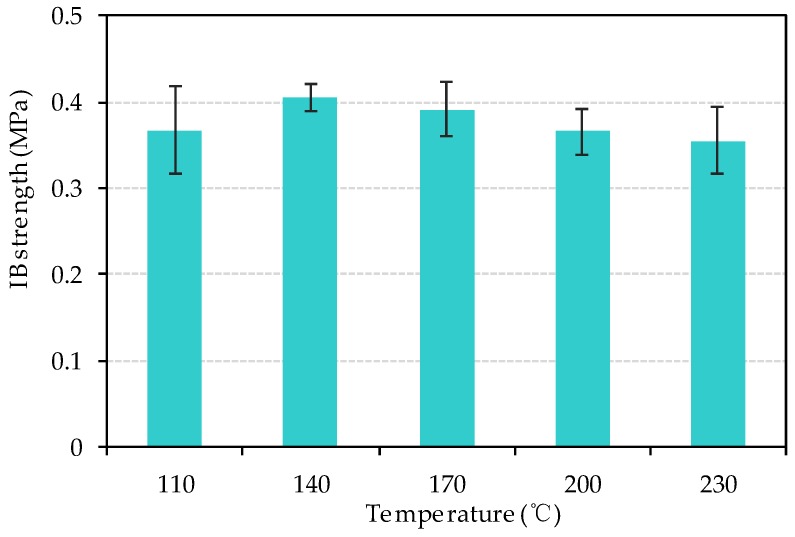
IB strength of bio-board at different temperature (*p* > 0.05).

The IB strength, also called tensile strength perpendicular to the faces, was an indispensable parameter for the fiberboard to evaluate whether the board would delaminate during post-treatment or not. The parameter was also related to the extent of bonding ability among fibers. Figure 11 represents the IB strength of bio-board in this study. The IB strength was between 0.37 MPa and 0.41 MPa.

At 110°C, the moisture content was up to 12.5%. They were filled with free water molecules among fibers, and it effected a combination among fibers. At 200 °C and 230 °C, high temperature brought about the transition of fiber properties. Therefore, the high moisture content and high temperature might cause the reduction of IB strength. According to JIS A5905, hardboard required minimum IB strength of 0.4 MPa. The produced bio-boards basically could not meet the requirements for this parameter.

**Figure 12:**
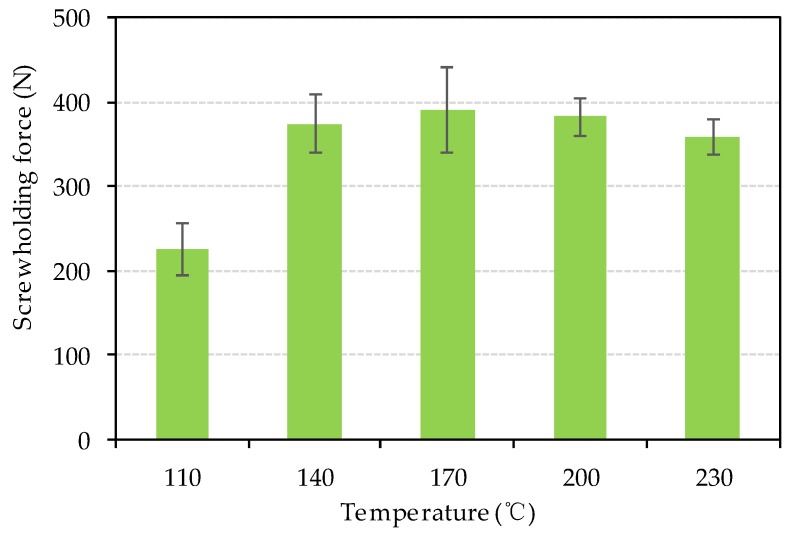
Screw-holding force of bio-board at different temperature (*p* < 0.05).

The fiberboard used in furniture and construction often requires screw fastening installation, so it is significant to evaluate the screw-holding force of bio-board. Figure 12 represents the screw-holding force of bio-board. The screw-holding forces were between 224.9 N and 390.4 N. The screw-holding force of bio-boards were greater than 350 N, except for that made at heating temperature of 110 °C. Generally, the screw-holding forces were similar to the bending strength as described above.

### 3.6. The Dimensional Stability Performance

**Figure 13:**
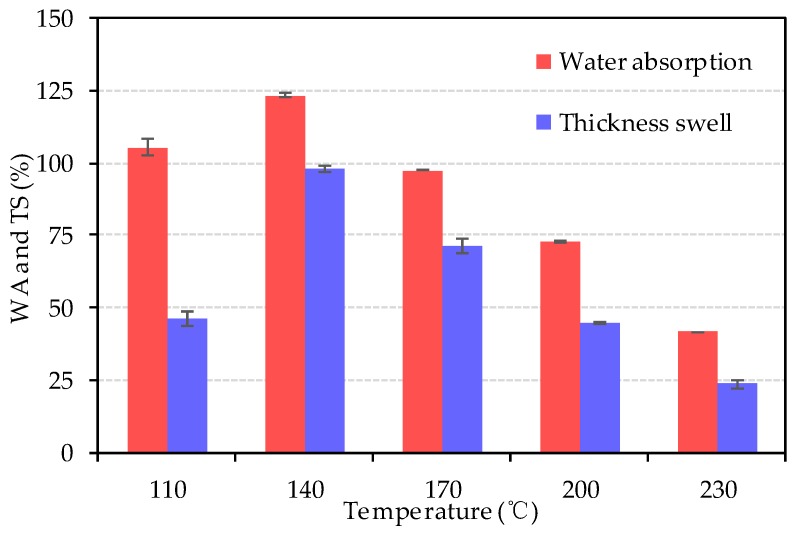
The WA and TS of bio-board at different temperatures (*p* < 0.05).

The results of WA and TS are shown in Figure 13. From 110 °C to 140 °C, the WA increased from 105.4% to 123.4% first, and then decreased from 123.4% to 41.5% after 140 °C. Similarly, the TS increased from 46.1% to 97.8%, then decreased from 97.8% to 23.5%. The dimensional stability performance of bio-board increased from 140 °C to 230 °C. As described in JIS A5908, the WA of hardboard S35 type should not be greater than 35%, and none of the bio-board could met the JIS standard for this parameter.

Both WA and TS increased during the heating temperature changing from 110 °C to 140 °C. This phenomenon might be caused by the following two reasons. First, the interspace among fibers of bio-board pressed at 110 °C had higher moisture content as shown in Figure 8; therefore, it was difficult to absorb more water than that of the bio-board pressed at 140 °C. Secondly, the bio-board was pressed at 140 °C with a higher density than that of 110 °C, and fibers huddle together tightly. After the WA test, the hydrogen bonds among the fibers opened, and the fiber swelled and the voids among fibers expanded significantly. Therefore, the bio-board pressed at 140 °C could absorb more water than that at 110 °C, and the TS rate was changed more obviously.

From 140 °C to 230 °C, WA and TS gradually decreased synchronously. The phenomenon could be attributed to the occurrence of the following chemical reactions. The first reason was the pyrolysis of hemicellulose. Owing to poor thermal stability of hemicellulose, the pyrolysis would first appear with the increase of heating temperature. Moreover, hemicellulose was a hydrophilic substance, and its reduction would improve the waterproof properties to some extent. The second reason was the condensation reaction of lignin. When the heating temperature was above 200 °C, the condensation reaction of lignin occurs, and the substance produced by the condensation reaction was black and hardly soluble in water, so the moisture content was greatly reduced after the water-soaking test.

## 4. Conclusions

In this study, bio-boards were made by using soybean straw without any petroleum-based resins. Moreover, the influence of heating temperature on mechanical properties and dimensional stability performance of bio-board was investigated. Some conclusions were revealed as follows:

In general, with the increase in temperature, there was a decrease in moisture content, the softening of lignin, and the pyrolysis of hemicellulose, which is beneficial to the improvement of the mechanical properties of the bio-board. However, the excessive heating temperature, especially at 230 °C, does not significantly promote improvement to most mechanical properties. On the other hand, the dimensional stability of the bio-board is greatly improved from 140 °C to 230 °C.

Increasing the heating temperature could improve the properties of bio-board to a certain extent. In future studies, different fiber lengths and different kinds of agriculture fiber could be used to produce bio-board, and investigate the effect of temperatures on the properties of the bio-board in order to derive a thorough explanation of the temperature effect on the properties of bio-board.

## References

[1] Li X., Li Y., Zhong Z., Wang D., Ratto J.A., Sheng K., Sun X.S. (2009). Mechanical and water soaking properties of medium density fiberboard with wood fiber and soybean protein adhesive. Bioresour. Technol..

[2] Kim S. (2009). Environment-friendly adhesives for surface bonding of wood-based flooring using natural tannin to reduce formaldehyde and TVOC emission. Bioresour. Technol..

[3] FAOSTAT, F.A.O 2019, FAOSTAT statistical database. http://www.fao.org/faostat/en/#data/QC.

[4] Cabrera E., Muñoz M.J., Martín R., Caro I., Curbelo C., Díaz A.B. (2015). Comparison of industrially viable pretreatments to enhance soybean straw biodegradability. Bioresour. Technol..

[5] Reddy N., Yang Y. (2009). Natural cellulose fibers from soybean straw. Bioresour. Technol..

[6] Hubbe M.A. (2006). Bonding between cellulosic fibers in the absence and presence of dry-strength agents–A review. BioResources.

[7] Geng X., Zhang S.Y., Deng J. (2006). Alkaline treatment of black spruce bark for the manufacture of binderless fiberboard. J. Wood Chem. Technol..

[8] Kusumah S.S., Umemura K., Yoshioka K., Miyafuji H., Kanayama K. (2016). Utilization of sweet sorghum bagasse and citric acid for manufacturing of particleboard I: Effects of pre-drying treatment and citric acid content on the board properties. Ind. Crop. Prod..

[9] Henao E.M., Quintana G.C., Ogunsile B.O. (2014). Development of binderless fiberboards from steam-exploded and oxidized oil palm wastes. BioResources.

[10] Halvarsson S., Edlund H., Norgren M. (2009). Manufacture of non-resin wheat straw fibreboards. Ind. Crop. Prod..

[11] Li X., Cai Z., Horn E., Winandy J.E. (2011). Effect of oxalic acid pretreatment of wood chips on manufacturing medium-density fiberboard. Holzforschung.

[12] Euring M., Rühl M., Ritter N., Kües U., Kharazipour A. (2011). Laccase mediator systems for eco-friendly production of medium-density fiberboard (MDF) on a pilot scale: Physicochemical analysis of the reaction mechanism. Biotechnol. J..

[13] Zhu X., Han S., Liu Y., Chen G. (2017). Effects of laccase incubated from white rot fungi on the mechanical properties of fiberboard. J. For. Res..

[14] Nasir M., Gupta A., Beg M.D.H., Chua G.K., Jawaid M., Kumar A., Khan T.A. (2013). Fabricating eco-friendly binderless fiberboard from laccase-treated rubber wood fiber. BioResources.

[15] Kumar S., Mohanty A.K., Erickson L., Misra M. (2009). Lignin and its applications with polymers. J. Biobased Mater. Bioenergy.

[16] Anglès M.N., Ferrando F., Farriol X., Salvadó J. (2001). Suitability of steam exploded residual softwood for the production of binderless panels. Effect of the pre-treatment severity and lignin addition. Biomass Bioenerg..

[17] Domínguez-Robles J., Tarrés Q., Delgado-Aguilar M., Rodríguez A., Espinach F.X., Mutjé P. (2018). Approaching a new generation of fiberboards taking advantage of self lignin as green adhesive. Int. J. Biol. Macromol..

[18] Zhou X., Tang L., Zhang W., Lv C., Zheng F., Zhang R., Du G.B., Tang B.J., Liu X. (2011). Enzymatic hydrolysis lignin derived from corn stover as an intrinstic binder for bio-composites manufacture: Effect of fiber moisture content and pressing temperature on boards’properties. BioResources.

[19] Okuda N., Hori K., Sato M. (2006). Chemical changes of kenaf core binderless boards during hot pressing (I): Influence of the pressing temperature condition. J. Wood Sci..

[20] Hashim R., Said N., Lamaming J., Baskaran M., Sulaiman O., Sato M., Sugimoto T. (2011). Influence of press temperature on the properties of binderless particleboard made from oil palm trunk. Mater. Des..

[21] Salvadó J., Velásquez J.A., Ferrando F. (2003). Binderless fiberboard from steam exploded Miscanthus sinensis: Optimization of pressing and pretreatment conditions. Wood Sci. Technol..

[22] Wang B., Li D.L., Chen T.Y., Qin Z.Y., Peng W.X., Wen J.L. (2017). Understanding the mechanism of self-bonding of bamboo binderless boards: Investigating the structural changes of lignin macromolecule during the molding pressing process. BioResources.

[23] (2003). Japanese Industrial Standards.

[24] Macedo J.S., Otubo L., Ferreira O.P., de Fátima Gimenez I., Mazali I.O., Barreto L.S. (2008). Biomorphic activated porous carbons with complex microstructures from lignocellulosic residues. Microporous Mesoporous Mat..

[25] Gurung M., Adhikari B.B., Kawakita H., Ohto K., Inoue K., Alam S. (2012). Selective recovery of precious metals from acidic leach liquor of circuit boards of spent mobile phones using chemically modified persimmon tannin gel. Ind. Eng. Chem. Res..

[26] Figen A.K., Terzi E., Yilgör N., Kartal S.N., Piskin S. (2013). Thermal degradation characteristic of Tetra Pak panel boards under inert atmosphere. Korean J. Chem. Eng..

[27] Junior C.P.A., Coaquira C.A.C., Mattos A.L.A., de Souza M.D.S.M., de Andrade Feitosa J.P., de Morais J.P.S., de Freitas Rosa M. (2018). Binderless fiberboards made from unripe coconut husks. Waste Biomass Valorization.

[28] Bledzki A.K., Mamun A.A., Volk J. (2010). Physical, chemical and surface properties of wheat husk, rye husk and soft wood and their polypropylene composites. Compos. Part A Appl. Sci. Manuf..

[29] Ramos D., El Mansouri N.E., Ferrando F., Salvadó J. (2018). All-lignocellulosic Fiberboard from Steam Exploded Arundo Donax L.. Molecules.

[30] Jakes J.E., Hunt C.G., Zelinka S.L., Ciesielski P.N., Plaza N.Z. (2019). Effects of Moisture on Diffusion in Unmodified Wood Cell Walls: A Phenomenological Polymer Science Approach. Forests.

[31] Stelte W., Clemons C., Holm J.K., Ahrenfeldt J., Henriksen U.B., Sanadi A.R. (2011). Thermal transitions of the amorphous polymers in wheat straw. Ind. Crops Prod..

